# Impact of biometric measurement error on identification of small‐ and large‐for‐gestational‐age fetuses

**DOI:** 10.1002/uog.21909

**Published:** 2020-01-08

**Authors:** D. Wright, A. Wright, E. Smith, K. H. Nicolaides

**Affiliations:** ^1^ Institute of Health Research University of Exeter Exeter UK; ^2^ Ultrasound Clinic Bovenmaas Rotterdam The Netherlands; ^3^ Harris Birthright Research Centre for Fetal Medicine King's College Hospital London UK

**Keywords:** estimated fetal weight, fetal abdominal circumference, fetal femur length, fetal growth restriction, fetal head circumference, large‐for‐gestational age, macrosomia, measurement error, small‐for‐gestational age

## Abstract

**Objectives:**

First, to obtain measurement‐error models for biometric measurements of fetal abdominal circumference (AC), head circumference (HC) and femur length (FL), and, second, to examine the impact of biometric measurement error on sonographic estimated fetal weight (EFW) and its effect on the prediction of small‐ (SGA) and large‐ (LGA) for‐gestational‐age fetuses with EFW < 10^th^ and > 90^th^ percentile, respectively.

**Methods:**

Measurement error standard deviations for fetal AC, HC and FL were obtained from a previous large study on fetal biometry utilizing a standardized measurement protocol and both qualitative and quantitative quality‐control monitoring. Typical combinations of AC, HC and FL that gave EFW on the 10^th^ and 90^th^ percentiles were determined. A Monte‐Carlo simulation study was carried out to examine the effect of measurement error on the classification of fetuses as having EFW above or below the 10^th^ and 90^th^ percentiles.

**Results:**

Errors were assumed to follow a Gaussian distribution with a mean of 0 mm and SDs, obtained from a previous well‐conducted study, of 6.93 mm for AC, 5.15 mm for HC and 1.38 mm for FL. Assuming errors according to such distributions, when the 10^th^ and 90^th^ percentiles are used to screen for SGA and LGA fetuses, respectively, the detection rates would be 78.0% at false‐positive rates of 4.7%. If the cut‐offs were relaxed to the 30^th^ and 70^th^ percentiles, the detection rates would increase to 98.2%, but at false‐positive rates of 24.2%. Assuming half of the spread in the error distribution, using the 10^th^ and 90^th^ percentiles to screen for SGA and LGA fetuses, respectively, the detection rates would be 86.6% at false‐positive rates of 2.3%. If the cut‐offs were relaxed to the 15^th^ and 85^th^ percentiles, respectively, the detection rates would increase to 97.0% and the false‐positive rates would increase to 6.3%.

**Conclusions:**

Measurement error in fetal biometry causes substantial error in EFW, resulting in misclassification of SGA and LGA fetuses. The extent to which improvement can be achieved through effective quality assurance remains to be seen but, as a first step, it is important for practitioners to understand how biometric measurement error impacts the prediction of SGA and LGA fetuses. © 2019 The Authors. *Ultrasound in Obstetrics & Gynecology* published by John Wiley & Sons Ltd on behalf of the International Society of Ultrasound in Obstetrics and Gynecology.

**CONTRIBUTION**

*What are the novel findings of this work?*

This study examines the impact of measurement errors in fetal head circumference, abdominal circumference and femur length on sonographic estimated fetal weight (EFW) and their effect on the prediction of small‐ (SGA) and large‐ (LGA) for‐gestational‐age fetuses.

*What are the clinical implications of this work?*

Measurement error in fetal biometry causes substantial error in EFW, resulting in misclassification of SGA and LGA fetuses. The extent to which improvement can be achieved through effective quality assurance remains to be seen but, as a first step, it is important for practitioners to understand how biometric measurement error impacts on the prediction of SGA and LGA fetuses.

## INTRODUCTION

Small‐for‐gestational‐age (SGA) neonates are at increased risk of stillbirth and adverse perinatal outcome^1^, ^2^, ^3^, ^4^. The expectation that these risks can potentially be reduced by medical interventions, such as early delivery, has led to the implementation of prenatal strategies for the identification of SGA fetuses. National guidelines from many developed countries define fetal growth restriction on the basis of ultrasonographic estimated fetal weight (EFW) < 10^th^ percentile and provide recommendations on monitoring and criteria for delivery of such pregnancies^5^. About 85% of SGA neonates are born at term^6^ and there is now good evidence that the predictive performance for a term SGA neonate is higher if, first, the method of screening is routine third‐trimester ultrasonographic fetal biometry rather than selective ultrasonography based on maternal risk factors and serial measurements of symphysis–fundus height^7^, and, second, the routine scan is carried out at 35 + 0 to 36 + 6 weeks' gestation rather than at 31 + 0 to 33 + 6 weeks^8^, ^9^. Similarly, large‐for‐gestational‐age (LGA) neonates with birth weight > 90^th^ percentile are at increased risk of perinatal death, birth injury and adverse neonatal outcome^2^, ^4^, ^10^, ^11^. Such risks could potentially be reduced by elective Cesarean section or early induction of labor to reduce the inevitable increase in fetal size with advancing gestational age^12^, ^13^, ^14^. As in cases of a SGA neonate, the best prediction of a LGA neonate is achieved by universal sonographic fetal biometry at 35 + 0 to 36 + 6 weeks' gestation^7^, ^15^.

The most widely adopted model for estimation of fetal weight is the one published by Hadlock *et al*. in 1985^16^, which combines ultrasonographic measurements of fetal abdominal circumference (AC), head circumference (HC) and femur length (FL) in the formula: Log_10_ (weight)  = 1.326 − 0.00326 × AC × FL + 0.0107 × HC + 0.0438  × AC.

A systematic review, which identified 46 studies describing a total of 70 models for EFW using various fetal measurements, found the model of Hadlock *et al*.^16^ to be the most accurate in predicting the weight of neonates born within 48 h after the scan^17^. However, data from implementation of a routine scan in 45 847 singleton pregnancies at 35 + 0 to 36 + 6 weeks' gestation showed that screening by sonographic EFW < 10^th^ percentile predicted only 70% of neonates with birth weight < 10^th^ percentile born within 2 weeks after assessment and about 45% of those born at any stage after assessment^18^. Similarly, EFW > 90^th^ percentile predicted only 71% of neonates with birth weight > 90^th^ percentile born within 10 days after the scan and 46% of those born at ≥ 37 weeks' gestation^15^. Possible explanations for the performance of EFW being only modest include, first, that some fetuses with EFW > 10^th^ percentile at the time of the scan may become SGA in the subsequent weeks before birth and some of those with EFW < 90^th^ percentile at the time of the scan may become LGA in the subsequent weeks before birth, and, second, that the measurements of AC, HC and FL used in the formula for EFW are imprecise.

The objectives of this study were, first, to obtain measurement‐error models for biometric measurements of AC, HC and FL, and, second, to examine the impact of biometric measurement error on EFW and its effect on the prediction of SGA and LGA fetuses or neonates.

## METHODS

We describe the methodology for examining the effect on EFW of errors in ultrasound measurements of AC, HC and FL taken at 36 + 0 weeks' gestation. At this gestational age, the 10^th^ and 90^th^ percentiles of the EFW distribution are 2453 g and 3086 g, respectively^19^. There are many combinations of measurements of AC, HC and FL that will provide these estimates. In order to obtain typical combinations, we found median biometry measurements contributing to EFW at earlier and later gestational ages^20^; at 34 + 3 weeks' gestation, the median values of AC (306.5 mm), HC (316.7 mm) and FL (65.8 mm) produced an EFW of 2453 g, and at 37 + 5 weeks, the median values of AC (333.3 mm), HC (330.3 mm) and FL (70.9 mm) produced an EFW of 3086 g. These combinations of measurements were used as references against which we could assess the effect of biometric measurement errors.

A multicenter study reported on measurements of fetal AC, HC and FL for 20 313 ultrasound images obtained prospectively from 4321 fetuses at 14–41 weeks' gestation; fetal AC, HC and FL were measured in a blinded fashion in triplicate on separately generated images^21^. We used data from this study^21^, which gives 95% limits of agreement for interobserver biometric measurements, to produce estimates of the standard deviations (SD) of individual measurement errors from the true dimension, as described in Appendix S1. Assuming a Gaussian distribution, we chose to examine the effects of errors of ± 0.67 and ± 1.64 SD; under the assumption of a Gaussian distribution, errors with magnitude greater than 0.67 SD occur in 50% of measurements and those with magnitude greater than 1.64 occur in 10% of measurements. Results are presented as EFW and EFW percentiles^18^ obtained by addition of the errors to the true measurements of AC, HC and FL outlined above.

To examine the effect of measurement error on the performance of prediction of SGA and LGA, we used a Monte‐Carlo simulation approach. The aim was to explore the effect of measurement error on the classification of fetuses as having EFW < 10^th^ or > 90^th^ percentile. For true percentiles between 0% and 100%, we obtained the proportion of observed EFWs that were < 10^th^ percentile and the proportion of observed EFWs > 90^th^ percentile. When screening for SGA, in a situation with no measurement error, this should be 100% if the true percentile is < 10% and 0% if the true percentile is ≥ 10%. If the true EFW is substantially lower than the 10^th^ percentile and the measurement error results in an EFW ≥ 10^th^ percentile, then the effect of measurement error would be to miss cases of SGA. Conversely, if the true EFW is substantially higher than the 10^th^ percentile and the measurement error results in an EFW < 10^th^ percentile, the effect of measurement error would lead to false positives. Similarly, when screening for LGA, in a situation with no measurement error, this should be 100% if the true percentile is > 90% and 0% if the true percentile is ≤ 90%. If the true EFW is substantially higher than the 90^th^ percentile and the measurement error results in an EFW ≤ 90^th^ percentile, then the effect of measurement error would be to miss cases of LGA. Conversely, if the true EFW is substantially lower than the 90^th^ percentile and the measurement error results in an EFW > 90^th^ percentile, the effect of measurement error would lead to false positives. Taking this one step further, we examined how the specification of percentile cut‐off affects the performance of screening for EFW < 10^th^ and > 90^th^ percentiles.

For each of the true percentiles, we obtained the gestational age for which the median values of AC, HC and FL^20^ gave the EFW corresponding to the true percentile as described above for the 10^th^ and 90^th^ percentiles. We then added random errors from an independent Gaussian distribution with SDs obtained from Cavallaro *et al*.^21^ to create a sample of 100 000 observed EFWs and computed the proportion that were < 10^th^ and > 90^th^ percentiles at 36 + 0 weeks. To explore the effect of improving measurement precision, we present results using the SDs obtained from the study of Cavallaro *et al*.^21^ and for these SDs reduced to 50% of their original value.

We also explored screening performance for SGA and LGA using various percentile cut‐offs.

The statistical software package R was used for data analysis^22^.

## RESULTS

**Figure 1:**
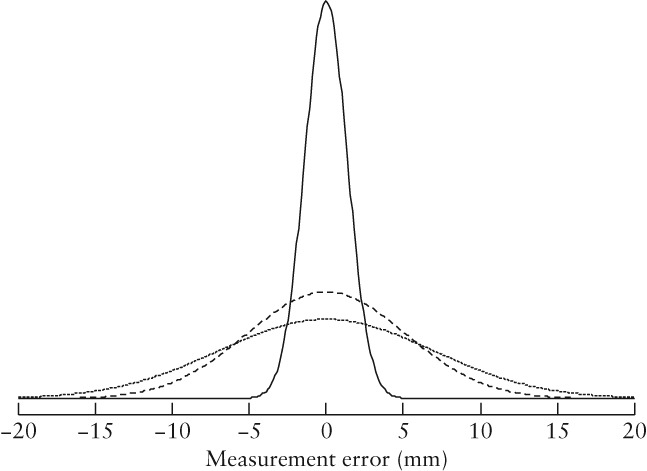
Error distribution of ultrasound measurements of fetal femur length (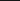), head circumference (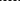) and abdominal circumference (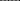).

The estimated SDs of errors in AC, HC and FL obtained from Cavallaro *et al*.^21^, using the method described in Appendix S1, were 6.93 mm for AC, 5.15 mm for HC and 1.38 mm for FL. The corresponding Gaussian distributions of these errors in measurements are shown in Figure 1; FL has the smallest spread and AC has the largest spread.

Table 1 shows the effect of different combinations of errors in biometry measures on EFW and EFW percentile for true EFWs on the 10^th^ and 90^th^ percentiles. If at 36 + 0 weeks' gestation there were no errors in the measurements of 306.5 mm for AC, 316.7 mm for HC and 65.8 mm for FL, EFW would be 2453 g, which corresponds to the 10^th^ percentile. An error resulting in underestimation in the measurement of AC by 11.4 mm (−1.64 SD), with no error in HC or FL, would result in EFW on the 2.6 percentile (2314 g). In contrast, overestimation of AC by 11.4 mm (+ 1.64 SD), would result in EFW of 2601 g, which corresponds to the 26.5 percentile. Overestimation of AC, HC and FL by 1.64 SD would result in EFW of 2732 g, which corresponds to the 46.9 percentile; overestimation by 0.67 SD would shift EFW to the 21.6 percentile. Conversely, if all measurements are subject to underestimation errors of 1.64 SDs, then EFW would shift from the 10^th^ to the 0.6 percentile, and, with underestimation by 0.67 SD, EFW would be shifted to the 3.7 percentile. Similarly, if at 36 + 0 weeks' gestation there were no errors in the measurements of 333.3 mm for AC, 330.3 mm for HC and 70.9 mm for FL, EFW would be 3086 g, which corresponds to the 90^th^ percentile. An error resulting in underestimation in the measurement of AC by 11.4 mm (−1.64 SD), with no error in HC or FL, would result in EFW on the 75.1 percentile (2923 g). In contrast, overestimation of AC by 11.4 mm (+ 1.64 SD) would result in EFW of 3257 g, which corresponds to the 97.0 percentile. Overestimation of AC, HC and FL by 1.64 SD would result in EFW of 3406 g, which corresponds to the 99.1 percentile; overestimation by 0.67 SD would shift EFW to the 95.9 percentile. Conversely, if all measurements are subject to underestimation by 1.64 SD, then EFW would shift from the 90^th^ to the 55.3 percentile, and, with underestimation by 0.67 SD, the EFW would be shifted to the 79.3 percentile.

**Table 1:** Effect of different combinations of error in fetal biometry measurements on estimated fetal weight (EFW) and EFW percentile, for true EFW of 10^th^ and 90^th^ percentiles

Error in multiples of SD (mm)			Small‐for‐gestational age		Large‐for‐gestational age	
AC	HC	FL	EFW (g)	EFW percentile	EFW (g)	EFW percentile
0.0	0.0	0.0	2453	10.0	3086	90.0
−1.64 (−11.4)	0.0	0.0	2314	2.6	2923	75.1
−0.67 (−4.6)	0.0	0.0	2395	6.1	3018	84.9
0.67 (4.6)	0.0	0.0	2513	15.5	3155	93.7
1.64 (11.4)	0.0	0.0	2601	26.5	3257	97.0
0.0	−1.64 (−8.5)	0.0	2403	6.5	3022	85.3
0.0	−0.67 (−3.5)	0.0	2432	8.4	3059	88.2
0.0	0.67 (3.5)	0.0	2474	11.8	3112	91.6
0.0	1.64 (8.5)	0.0	2505	14.7	3150	93.5
0.0	0.0	−1.64 (−2.3)	2380	5.3	3007	84.0
0.0	0.0	−0.67 (−0.9)	2423	7.8	3053	87.8
0.0	0.0	0.67 (0.9)	2484	12.7	3118	91.9
0.0	0.0	1.64 (2.3)	2529	17.3	3166	94.2
−1.64 (−11.4)	−1.64 (−8.5)	−1.64 (−2.3)	2194	0.6	2784	55.3
−0.67 (−4.6)	−0.67 (−3.5)	−0.67 (−0.9)	2345	3.7	2960	79.3
0.67 (4.6)	0.67 (3.5)	0.67 (0.9)	2565	21.6	3214	95.9
1.64 (11.4)	1.64 (8.5)	1.64 (2.3)	2732	46.9	3406	99.1

AC, abdominal circumference; FL, femur length; HC, head circumference.

**Figure 2:**
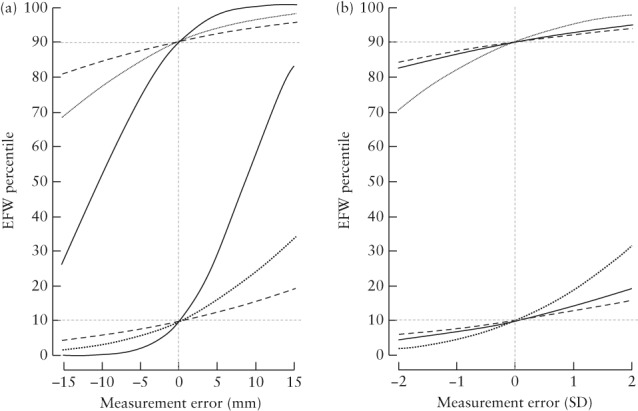
Effect of error in individual biometry measurements in mm (a) and SD units (b) on estimated fetal weight (EFW) percentile at 36 + 0 weeks' gestation, if true EFW is on 90^th^ percentile (upper lines) or 10^th^ percentile (lower lines), for femur length (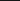), head circumference (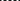) and abdominal circumference (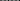). Zero error in remaining two components is assumed for each curve.

The effect of errors in individual biometry measurements, in mm and SD units, on EFW at 36 + 0 weeks' gestation when true EFW is on the 10^th^ and 90^th^ percentiles (2453 g and 3086 g, respectively), with corresponding biometry measurements as outlined above, can be seen in Figure 2. In terms of SD units, which allow for a fair comparison of the three measures, errors in AC have the largest impact on EFW percentile and errors in HC have the smallest impact on EFW percentile.

Table 2 shows the performance of screening for SGA and LGA neonates for various percentile cut‐offs, when EFW is obtained using AC, HC and FL subject to random Gaussian errors with means of 0 mm and SD according to Cavallaro *et al*.^21^. Assuming errors according to such distributions, when the 10^th^ and 90^th^ percentiles are used to screen for SGA and LGA fetuses, respectively, the detection rates would be 78.0% at false‐positive rates of 4.7%. If the cut‐offs were relaxed to the 30^th^ and 70^th^ percentiles, the detection rates would increase to 98.2%, but at false‐positive rates of 24.2%. Assuming half of the spread in the error distribution, when the 10^th^ and 90^th^ percentiles are used to screen for SGA and LGA fetuses, respectively, the detection rates would be 86.6% at false‐positive rates of 2.3% and, if the cut‐offs were relaxed to the 15^th^ and 85^th^ percentiles, respectively, the detection rates would increase to 97.0% and the false‐positive rates would increase to 6.3%.

**Table 2:** Performance of screening for small‐ (SGA) and large‐ (LGA) for‐gestational‐age neonates for various estimated fetal weight (EFW) percentile cut‐offs, when EFW is obtained by abdominal circumference, head circumference and femur length, subject to random Gaussian errors with means of 0 mm and estimated SDs of errors according to Cavallaro *et al.* ^21^ *,* and after reduction of these errors by 50%

EFW percentile cut‐off for:		DR	FPR	PPV	100 – NPV	*n* based on population of 100 000			
SGA	LGA	(%)	(%)	(%)	(%)	TP	FP	FN	TN
SDs as per Cavallaro *et al*.									
10^th^	90^th^	78.0	4.7	64.7	2.5	7800	4250	2200	85 750
15^th^	85^th^	87.9	9.6	50.5	1.5	8790	8611	1210	81 389
20^th^	80^th^	94.4	14.6	41.9	0.7	9440	13 101	560	76 899
25^th^	75^th^	97.4	19.7	35.5	0.4	9740	17 692	260	72 308
30^th^	70^th^	98.2	24.2	31.1	0.3	9820	21 772	180	68 228
35^th^	65^th^	99.2	29.2	27.4	0.1	9920	26 303	80	63 697
40^th^	60^th^	99.4	34.3	24.4	0.1	9940	30 863	60	59 137
45^th^	55^th^	99.7	39.6	21.8	0.0	9970	35 664	30	54 336
50^th^	50^th^	100.0	43.6	20.3	0.0	10 000	39 274	0	50 726
SDs reduced by 50%									
10^th^	90^th^	86.6	2.3	80.9	1.5	8660	2040	1340	87 960
15^th^	85^th^	97.0	6.3	62.9	0.4	9700	5711	300	84 289
20^th^	80^th^	99.7	11.6	48.8	0.0	9970	10 461	30	79 539
25^th^	75^th^	100.0	16.7	40.0	0.0	10 000	15 012	0	74 988
30^th^	70^th^	100.0	22.4	33.1	0.0	10 000	20 192	0	69 808
35^th^	65^th^	100.0	28.0	28.4	0.0	10 000	25 193	0	64 807
40^th^	60^th^	100.0	33.5	24.9	0.0	10 000	30 123	0	59 877
45^th^	55^th^	100.0	38.5	22.4	0.0	10 000	34 654	0	55 346
50^th^	50^th^	100.0	44.1	20.1	0.0	10 000	39 664	0	50 336

DR, detection rate; FN, false negative; FP, false positive; FPR, false‐positive rate; NPV, negative predictive value; PPV, positive predictive value; TN, true negative; TP, true positive.

**Figure 3:**
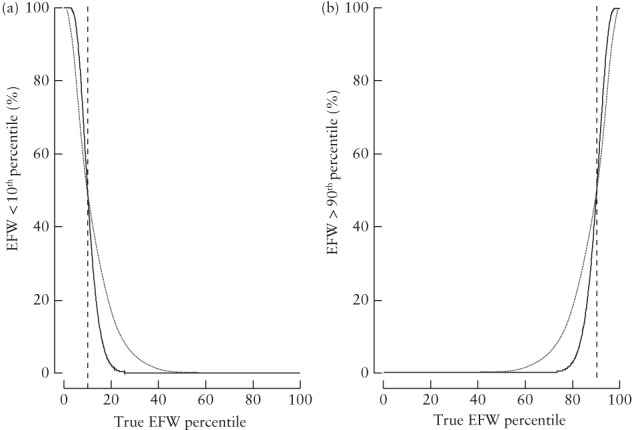
Proportion of fetuses with estimated fetal weight (EFW) < 10^th^ percentile (a) and > 90^th^ percentile (b) for true EFW percentiles from 0–100, with estimated SDs of errors (6.93 mm for abdominal circumference, 5.15 mm for head circumference and 1.38 mm for femur length) obtained from Cavallaro *et al*.^21^ (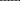) and with SDs of errors reduced by 50% (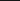).

Figure 3 shows the proportion of fetuses with EFW < 10^th^ and > 90^th^ percentiles for true percentiles from 0–100. On the basis of estimated SDs of errors obtained from Cavallaro *et al*.^21^, of those with EFW truly on the 10^th^ percentile, only 50% would be classified as SGA due to measurement error. Of those with true EFW on the 20^th^ percentile, approximately 16% would be classified as < 10^th^ percentile due to measurement error. Of those with true EFW on the 5^th^ percentile, approximately 20% would be classified as ≥ 10^th^ percentile due to measurement error. The performance of EFW < 10^th^ percentile improves with decreasing error variation. Similarly, due to the symmetry of the distribution of percentiles, of those with EFW truly on the 90^th^ percentile, only 50% would be classified as LGA due to measurement error. Of those with true EFW on the 80^th^ percentile, approximately 16% would be classified as > 90^th^ percentile. Of those with true EFW on the 95^th^ percentile, approximately 20% would be classified as ≤ 90^th^ percentile due to measurement error.

## DISCUSSION

### Main findings

The findings of this study demonstrate that, even with a comprehensive package of ultrasound quality control^21^ and use of the most widely accepted and accurate model for EFW^16^, ^17^, errors in measurements of fetal AC, HC and FL have a large impact on EFW and therefore EFW percentile. Even relatively small errors in a single component parameter can alter potential clinical decisions, with either an appropriate‐for‐gestational‐age (AGA) fetus being classified as SGA or LGA, or a SGA or LGA fetus being classified as AGA.

### Comparison with previous studies

There is extensive literature on the use of measurement‐error models such as the one we applied in this study^23^. We are not aware of other work in which error SDs have been extracted from limits of agreement. However, this is an application of standard distribution theory.

### Clinical implications

There is now good evidence that, first, about 85% of SGA neonates are born at term^6^, second, the best way to identify such SGA and LGA fetuses is by routine sonography at 35 + 0 to 36 + 6 weeks' gestation^6^, ^7^, ^8^, ^9^, ^15^, and, third, the most accurate model for assessment of EFW is that reported by Hadlock *et al*., which combines ultrasonographic measurements of fetal AC, HC and FL^16^, ^17^. However, as demonstrated in this study, measurement error in fetal biometry can cause substantial error in EFW, resulting in misclassification of both SGA and LGA neonates.

There are three potential approaches for improving the performance of prenatal prediction of SGA and LGA neonates. First, improving the models for assessment of EFW; but, despite many efforts in the last 50 years and the publication of more than 70 models, the one reported by Hadlock *et al*. in 1985 remains the most widely accepted and accurate one^16^, ^17^. Attempts at improving the prediction of birth weight by the addition of maternal characteristics to fetal biometry have not been found to be successful^24^, ^25^. Similarly, there is some contradictory evidence as to whether the precision of EFW can be improved by three‐dimensional ultrasound volumetry^26^, ^27^, ^28^. Second, development of a standardized fetal biometric ultrasound measurement protocol, involving training, assessment and certification of sonographers and both qualitative and quantitative quality‐control monitoring, can minimize systematic error and ensure high reproducibility^21^. As demonstrated in this study, it would be necessary to improve this process further to reduce errors in measurements and this could potentially be achieved by sonographers repeating measurements when EFW is near the cut‐off of interest, such as the 10^th^ or 90^th^ percentile. The third approach for potential improvement of the performance of prenatal prediction of adverse perinatal outcome in pregnancies undergoing routine ultrasound examination at 35 + 0 to 36 + 6 weeks' gestation is to accept the limitations of sonographic EFW at the cut‐offs of the 10^th^ and 90^th^ percentiles, respectively, and base clinical management, including serial scans, on an EFW cut‐off of the 40^th^ percentile together with findings of fetal Doppler indices for SGA fetuses^18^ and the 70^th^ percentile for LGA fetuses^15^.

### Strengths and limitations

Use of a Monte‐Carlo simulation approach allows examination of the effect of measurement error on EFW and clinical interpretation in the hypothetical situation in which the true biometric measurements are known. We can also explore the effect of different levels of variability on EFW, enabling us to set acceptable limits on the level of error variability.

Limitations are the assumptions of uncorrelated Gaussian distributed errors with constant SDs, centered on zero. In practice, the correlations are likely to be positive which will mean that the errors tend to be in the same direction, increasing their effect on EFW. The assumption that the errors are uncorrelated could therefore be considered as conservative. Another limitation is that, although there are many combinations of biometry that will result in an EFW on the 10^th^ and 90^th^ percentiles, we used median levels at an earlier gestational age for the former and those at a later gestational age for the latter. Consequently, our results apply to the situation in which the fetus has biometry consistent with an earlier gestational age for EFW on the 10^th^ percentile, and a later gestational age for EFW on the 90^th^ percentile. Standard deviations were obtained from a study with a comprehensive package of ultrasound quality control^21^. However, interobserver comparisons relate to caliper placement by different individuals using the same image; this ignores variations between images, leading to underestimation of error variation. In other settings, measurement‐error SDs may differ from those assumed here due to differences in populations, equipment, quality‐control procedures and other factors.

### Conclusions

Measurement error in fetal biometry causes substantial error in EFW, resulting in misclassification of SGA and LGA fetuses. This explains, to a certain extent, the limited performance of sonographic EFW in screening for SGA and LGA neonates. For reliable assessment of SGA and LGA by EFW, the biometric measurement‐error SDs obtained from Cavallaro *et al*.^21^ should be reduced by at least 50%. The extent to which improvement can be achieved through effective quality assurance remains to be seen but, as a first step, it is important for practitioners to understand how biometric measurement error impacts on the prediction of SGA and LGA fetuses.

## Supporting information


**Appendix S1** Calculation of error standard deviation
