# Supplementary material for: Impact of biometric measurement error on identification of small‐ and large‐for‐gestational‐age fetuses
Source: Ultrasound Obstet Gynecol. 2020 Jan 8;55(2):170–6. doi: 10.1002/uog.21909 (PMC7027772; doi:10.1002/uog.21909)
Supplement: Supplementary file 1 — Appendix S1 Calculation of error standard deviation [file UOG-55-170-s001.docx]

**Appendix S1** Calculation of error standard deviation

Given two biometric measurements $X_{1}$ and $X_{2}$, each assumed to be composed of a true dimension that we denote by μ and a random error, so that $X_{1}= \mu+\epsilon_{1}$ and $X_{2}= \mu+\epsilon_{2}$.

Where $\epsilon_{1}$ and $\epsilon_{2}$ are uncorrelated random errors with mean zero and standard deviation proportional to $\mu$ so that $Var\left[ X_{i} \right]=Var\left[ \epsilon_{i} \right]=\mu^{2}\sigma^{2}$.

With these assumptions $Var\left[ X_{1}-X_{2} \right]=2\mu^{2}\sigma^{2}$

Cavallaro *et al.*^21^ assessed the variability in measurement in terms of limits of agreement, where

$$LoA=100\times1.96 \sigma_{p}$$

where $\sigma_{p}^{2}$ is the variance of

$$\frac{X_{1}-X_{2}}{(X_{1}+X_{2})/2}$$

Approximation $(X_{1}+X_{2})/2$ in the denominator by $\mu$

$$Var\left[ \frac{X_{1}-X_{2}}{\left( X_{1}+X_{2} \right)/2} \right]\cong Var\left[ \frac{{(X}_{1}-X_{2})}{\mu} \right]=\frac{Var\left[ X_{1}-X_{2} \right]}{\mu^{2}}=2\sigma^{2}$$

Thus, we estimate the standard deviation of the measurement errors $\epsilon_{i}$ by $\mu\sigma_{p}/\sqrt{2}$ where $\sigma_{p}=LoA/196$.
